# A rare case of right coronary artery dissection during routine left heart catheterization

**DOI:** 10.21542/gcsp.2024.24

**Published:** 2024-04-20

**Authors:** Noreen Mirza, Vikramjit Purewal, Joanna Pater, Sayed A. Shah, Fajr Mirza, Iyad Farouji, Preet Randhawa

**Affiliations:** 1 Department of Internal Medicine, Saint Michael’s Medical Center, Newark, New Jersey, USA; 2 Department of Internal Medicine, Mountain Vista Medical Center, Mesa, Arizona, USA; 3 Medical Education Saint Michael’s Medical Center, Newark, New Jersey, USA; 4 Department of Cardiology, Saint Michael’s Medical Center, Newark, New Jersey, USA

## Abstract

Percutaneous coronary intervention (PCI) is an effective method for coronary revascularization, however, alongside its benefits, it can be accompanied by complications. Catheter induced coronary artery dissection (CICAD) is rare and the consequences can be devastating if left untreated. The incidence has been reported to be as low as 0.1%. Also, propagation of the dissection to the aortic root remains uncommon. The mechanism of dissection is related to mechanical injury to the arterial wall during manipulation with the catheter or wire. It may also occur due to injection of contrast, stenting or balloon dilation. Timely recognition is important in these cases to facilitate optimal patient outcomes which is usually accomplished with stenting. Herein, we report a rare case of a 68-year-old female with multivessel coronary artery disease who presented for routine left heart catheterization and developed catheter induced right coronary artery (RCA) dissection with propagation towards the aortic root which was treated with stenting and watchful waiting.

## Introduction

CICAD is a rare complication of cardiac catheterization. It is associated with several life-threatening outcomes including acute coronary syndrome, expansion into the coronary tree or towards the aortic root, and need for emergent vascular surgery. Several population studies have investigated the incidence of iatrogenic coronary artery dissection, however epidemiologic data is limited due to the relatively low prevalence of this condition.

Despite this, coronary artery dissection is reported as the most common indication for surgery following PCI^1^. Klaudel et al. estimated the incidence of CAD to be 0.126% of all catheterization procedures^2^. Mortality was at 4.2% for all dissections, and 6.25% if the dissection extended to the aortic root^2^. Other studies have found 0.09% incidence and 5.9% mortality^3^, or 0.003% incidence with 2.7% mortality^4^. Patient characteristics associated with higher rates of dissection included: female sex, hypertension, history of stroke, and prior kidney disease^2^. BMI and age had no correlation with dissection risk^2^.

The right and left main coronary arteries have comparable rates of injury^2^. Propagation of dissection is not uncommon, but rarely involves the aorta. Retrograde extension reaching the aortic root is reported at 0.008%–0.02% for diagnostic catheterizations and 0.06%–0.07% for PCI^5^, while aortic involvement resulting from extension is estimated to occur in 2.1% of cases^2^.

### Case presentation

A 68-year-old female with a medical history of multivessel coronary artery disease, diabetes mellitus, hypothyroidism, obesity, hypertension, obstructive sleep apnea and hyperlipidemia presented for cardiac catheterization due to chest pain and shortness of breath on exertion for the past few months.

**Figure 1.**
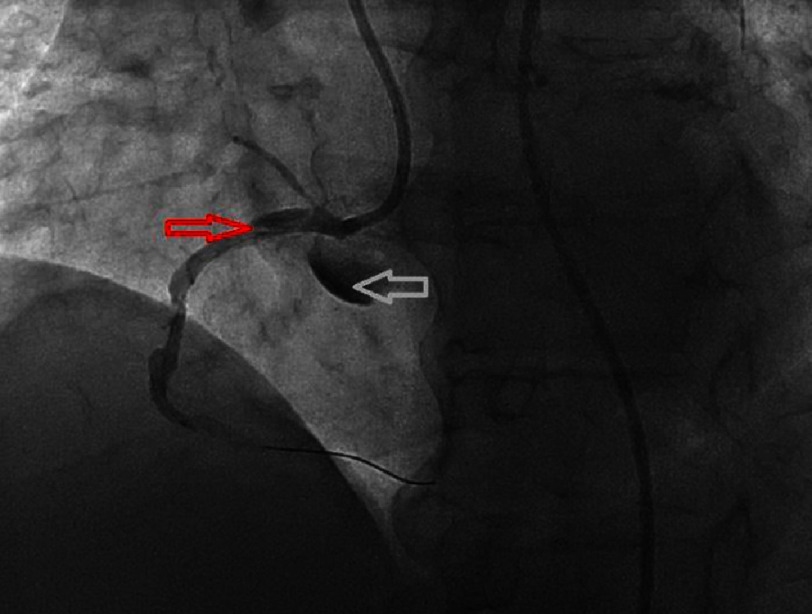
Left heart catheterization revealing the right coronary artery with dissection (red arrow) propagating towards the aortic root (white arrow).

On examination, her temperature was 97.7 °F, blood pressure was 162/98 mmHg, heart rate was 81 beats per minute and saturation was 97% on room air. Cardiac examination revealed normal S1/S2 and no murmurs, rubs or gallops. EKG was sinus rhythm with poor R wave progression, low voltage and no acute ST-T changes. Cardiac catheterization revealed a heavily calcified 90% lesion in the RCA. The RCA was engaged using a JR4 6 French guiding catheter intracoronary nitroglycerin and IV heparin was administered after that the lesion in the RCA was crossed with the help of a BMW wire. There were multiple attempts made to pass a 2.5 mm noncompliant balloon which were unsuccessful. The decision was made to proceed with rotational atherectomy of the RCA however, when the guidewire was removed dissection was visualized in the proximal portion of the RCA to the aortic cusp as seen in Figure 1.

The dissection in the RCA was crossed then intraluminal position was confirmed and the lesion was predilated using a two mm balloon. Due to the risks of hemodynamic instability, the proximal RCA was stented using a 3.5 × 15 mm drug eluting stent and a 3.0 × 12 mm drug-eluting stent which extended to the ostium of the RCA. The patient was transferred to the coronary care unit for closer monitoring and the patient remained hemodynamically stable and was discharged after a few days.

## Discussion

### Pathogenesis

As with most cases of dissection, catheter-induced coronary artery dissection primarily results from intimal injury. Any changes to the position of the catheter –including patient arm movements or catheter manipulation - can displace it and increase the risk of damage to the arterial wall^5^. Wedge contrast injection was the most identified mechanism, resulting in 46% of coronary dissections and 75% of spiral dissections as reported in a population study by Klaudel et al.^2^. Forceful catheter engagement (28% cases) and deep catheter insertion (26% cases) were also common mechanisms of injury^2,5^.

Several procedure characteristics increase the risk of dissection. Guiding catheters are associated with many dissection cases (85%) while diagnostic catheters contribute to only 15% of cases^2,3^. Deep catheter insertion, amplatz-shaped catheters, and forceful catheter engagement have also been implicated with this condition^6^. There has been speculation about access site contributing to the risk of CAD, but no significant data has been found to support this claim^7^. However, tortuosity of the vasculature is an independent risk factor for CAD regardless of access site^8^.

Propagation of dissection can occur in approximately 30.2% of cases, but most cases are self-limited and do not disrupt flow through the arterial lumen^2^. Vessel occlusion due to extension of a dissection is a serious and life-threatening complication requiring urgent stenting or surgery. Delayed stenting, repeat injections of contrast, and unchanged catheters are associated with continued expansion and more extensive vessel involvement^2^. Retrograde expansion of a dissection is rare but can progress up to the sinus of Valsalva and even involve the ascending aorta^5^. Interestingly, structures that protect against anterograde expansion–stent, atherosclerotic plaques, or arterial occlusion–can instead redirect contrast flow in the direction of the aorta and promote upstream dissection^2^. While the prevalence of retrograde dissection is not well-established, some research has suggested that the histological characteristics of the right coronary artery make it less resistant to traction and thus more prone to aortic involvement^9^.

The most important prognostic factor in CAD is the extent to which anterograde perfusion is preserved^3^. Degree of luminal obstruction correlates with mortality and clinical outcomes. Well-established collaterals can protect the myocardium from ischemic damage; thus prognosis is worse for dissected vessels with no preexisting stenosis^1^.

### Diagnosis

The clinical presentation of iatrogenic coronary artery dissection ranges from asymptomatic to acute coronary syndrome and cardiogenic shock. Coronary angiography is the first-line method for diagnosis. On imaging, an intraluminal flap can be visualized, accompanied by the appearance of multiple lumens and contrast staining of the arterial walls^6^. Intracoronary imaging offers a second-line option for diagnosis of uncertain cases. Dissection flaps, intimal tears, false lumens, and intramural hematoma can be identified in this manner. Intravascular ultrasound is useful to identify the entry point of dissection and confirm if the guidewire is in the true or false lumen^10^. IVUS can also be used to image the size and extent of the intramural hematoma^11^. While optical coherence tomography (OCT) offers better spatial resolution to visualize the affected wall, this method of imaging requires a high rate of contrast injection and is associated with increased intraluminal pressure, which can enlarge an existing dissection^10–12^. IVUS is generally considered safer as a result^12^. Retrograde aortic dissection can be diagnosed with CT scan or transesophageal echocardiography^10^.

### Classification

Classification of coronary artery dissections is based on angiographic findings. Type A and B dissections are generally benign and amenable to conservative management^3^. Both are characterized by radiolucency and minimal persistence of contrast. Type C and D dissections present with persistence of contrast in the extraluminal areas, with type D specifically showing evidence of spiral dissection. Types E and F are associated with filling defects, either partial (E) or complete (F). Type F dissections have the highest mortality and worst prognosis; however types C-F are all at risk for acute vessel closure and further complications^10^.

### Management

Management of coronary artery dissection is determined based on clinical severity. While low-grade, localized, and non-obstructing dissections often resolve spontaneously and can be managed conservatively, many catheter-induced dissections impair luminal flow to some degree and require PCI or surgical intervention^3^. Concerns have also arisen about scar formation and late-onset stenosis in patients treated conservatively^5^. In cases of acute vessel closure, urgent revascularization and re-establishment of perfusion is indicated. Signs of acute onset myocardial ischemia - including chest pain or new ECG changes - require intervention regardless of whether significant vessel obstruction is identified on imaging^10^.

Revascularization attempts can be made with percutaneous coronary intervention or coronary bypass graft surgery. Both interventions have considerable data supporting their safety and efficacy in treating CAD, however many cases can be successfully managed with stenting alone. An appropriate stent should be sufficiently long to seal off the dissection entry site and extend to an area of normal vessel, such that the intramural hematoma is fully compressed and not allowed an opportunity to propagate^10^. Calcified or tortuous vessels can impede the successful deployment of a stent^6^. Prognosis after PCI is favorable, with only 2% of cases proceeding to acute vessel re-occlusion as compared to 32% without stenting^6^. Hemodynamic instability and delays in restoration of blood flow to the affected regions are indications for surgery^5^.

Clinical indications for retrograde extension to the aorta are not well-established and must be made on a case-by-case basis. Aortic involvement can be managed conservatively if limited to the sinus of Valsalva, or by stenting of the dissection entry site to prevent further progression^13^. Extensive aortic lesions usually require surgery, however successful treatment has also been reported with stenting alone^13^. Hemodynamic instability and ischemia of the aortic branches require urgent surgical intervention^5^.
